# Extensor Tendon Rupture After Distal Radioulnar Joint Surgery: A Case Series

**DOI:** 10.7759/cureus.14118

**Published:** 2021-03-25

**Authors:** Connor Fletcher, Francis J Sirch, Daniel Fletcher, Jonas Matzon, Pedro Beredjiklian

**Affiliations:** 1 Division of Hand Surgery, Rothman Orthopaedic Institute, Philadelphia, USA; 2 Division of Orthopedics, Rothman Orthopaedic Institute, Philadelphia, USA

**Keywords:** extensor tendon rupture, tendon repair, druj osteoarthritis, druj, distal radioulnar joint

## Abstract

Symptomatic arthritis of the distal radioulnar joint (DRUJ) is often treated nonoperatively but with persistent symptoms may be treated surgically with partial or complete distal ulna resection. In many of these cases, ulna resection in combination with tendon reconstruction can successfully restore hand function. We identified three patients who underwent the Darrach procedure to treat DRUJ arthritis that developed attritional ruptures due to sharp prominent bone edges or dorsal capsule disruption. In addition to our recent three patients, an additional three isolated case reports, and two cases in a 29-patient series reported post-operative extensor tendon rupture as a complication after a Darrach procedure more than 30 years ago. While extensor tendon rupture is rarely reported in recent literature as a complication of distal ulna excision, surgeons may be able to minimize the risk of this complication intra-operatively by ensuring the resected distal ulnar stump is smooth, free of bony prominences, any capsular deficiencies are reconstructed, and that extensor tendons are able to glide freely.

## Introduction

Distal radioulnar joint (DRUJ) arthritis, whether due to degeneration, inflammation, or trauma, can be a source of substantial pain and functional impairment [1-3]. Typically, DRUJ arthritis can be treated non-operatively, but occasionally it may require surgical intervention after the failure of conservative treatment. Surgical treatment for DRUJ osteoarthritis often involves partial or complete distal ulna resection [4-7]. In some cases, DRUJ arthritis can lead to extensor tendon rupture with loss of digital extension as originally described by Vaughan-Jackson [8-11]. In these cases, distal ulna resection in combination with tendon reconstruction can successfully restore hand function. Numerous complications after distal ulna excision have been reported including infection, wound breakdown, stump impingement or instability, ulnar exostoses, extensor carpi ulnaris (ECU) tendonitis, and persistent pain [6,7,12]. We identified three patients who paradoxically developed extensor tendon ruptures after distal ulna excision.

## Case presentation

Case 1

An 81-year-old, right-hand-dominant male presented with right ulnar wrist pain. Pre-operative radiographs revealed end-stage DRUJ osteoarthritis without dorsal instability or subluxation. Following the failure of non-operative treatment, the patient underwent a Darrach procedure with ulnar stump stabilization utilizing the ECU and flexor carpi ulnaris (FCU) tendons. There were no complications until two months post-operatively, when the patient presented with an acute inability to actively extend his ring finger (RF) and small finger (SF). Clinically, the patient was diagnosed with an attritional rupture of his extensor tendons, which was confirmed by MRI. Between the time of consent to the scheduled extensor tendon reconstruction, the patient lost the ability to extend his long finger (LF).

**Figure 1:**
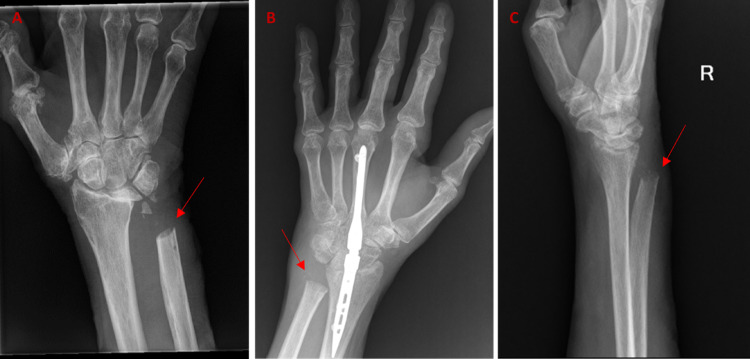
Sharp Edges of the Distal Ulna. (A) An 81-year-old male, a two-month post-operative image presenting with an inability to extend ring finger and small finger, was found to have extensor tendon rupture confirmed by MRI. Red arrows denote a sharp edge of the distal ulna in all images. (B) A 63-year-old female, two-month post-operative image, presenting with an inability to extend small finger and weak ring finger extension. (C) A 68-year-old female, four-week postoperative imaging, presenting with the loss of ring finger extension.

Upon surgical exploration, attritional ruptures of the LF, RF, and SF extensor digitorum communis (EDC) tendons were attributed to a prominent dorsal sharp edge of the distal ulna (Figure 1A). The extensor digitorum quinti (EDQ) remained intact but was diminutive. The prominent edge was rasped smooth and the dorsal capsule was reconstructed with local tissue. A flexor carpi radialis (FCR) to LF, RF, and SF EDC tendon transfer was performed with no intra-operative complications. A custom orthoplast orthosis maintaining the metacarpophalangeal joints of all fingers in extension was worn for six weeks to protect the extensor tendon reconstruction. Subsequently, the patient participated in hand therapy for three months and regained full active range of motion (ROM) of his wrist and fingers. The patient experienced no further complications and returned to full activities.

Case 2

A 63-year-old, left-hand-dominant female with radiocarpal and DRUJ osteoarthritis underwent a total wrist arthrodesis and Darrach procedure. The wrist arthrodesis was accomplished using an intramedullary implant with bone grafting. There were no complications until two months post-operatively when the patient returned to the clinic unable to extend her left SF and only weakly able to extend her RF. An MRI revealed LF, RF, and SF extensor tendon ruptures. Therefore, 10 weeks following her index procedure, the patient underwent exploration. Intra-operatively, the RF EDC, SF EDC, and EDQ were completely ruptured, and the LF EDC had a high-grade partial rupture. The index finger (IF) EDC and extensor indicis proprius (EIP) remained intact. The distal ulna was found to have a prominent sharp edge (Figure 1B), which appeared to be causing the attritional tendon ruptures. Following rasping of the prominent distal ulna and stabilization of the DRUJ with local tissue, side-to-side tendon transfers of the SF, RF, and LF EDC tendons to the IF EDC and EIP were performed using a pulvertaft weave. Post-operatively, the fingers were splinted in extension for five weeks, at which time hand therapy was initiated. The patient experienced no further complications and returned to full activities.

Case 3

A 68-year-old, right-hand-dominant retired female presented with three months of right dorsal wrist pain. She was diagnosed with severe right DRUJ arthritis with overlying extensor tenosynovitis. Given her presentation, there was a concern for impending extensor tendon attritional ruptures. Therefore, the patient elected to undergo a Darrach procedure with radical extensor tenosynovectomy. Upon exploration, the extensor tendons were intact without rupture. The distal ulna was excised and a capsular imbrication was performed in full supination to prevent distal ulna instability. At her two-week post-operative visit, the patient had good finger ROM; however, at four weeks, the patient returned to the office with a loss of active RF extension. Given the suspicion of attritional extensor tendon rupture, the patient was scheduled for surgical exploration and probable reconstruction under local anesthesia.

Intra-operatively, there was perforation of the DRUJ capsule, without a specific sharp bony prominence but the distal ulna was dorsally prominent with an oblique resection of the distal ulna (Figure 1C). The RF EDC and LF EDC were found to be ruptured. To prevent impingement, an additional distal ulna was removed. Moreover, the proximal ruptured tendon remnants were excised and woven over the dorsal DRUJ capsule to create a tight capsular closure with the hope that this would prevent irritation from the underlying distal ulna and provide a smooth gliding surface for tendon transfer. Finally, the LF EDC was woven into the IF EDC to give an adequate extension, and the RF EDC was transferred to the EDQ. The patient demonstrated a full active finger extension intra-operatively. Following closure, the patient was placed in a splint with the fingers in full extension. A custom-fitted orthosis was fabricated one-week after reconstruction but she was not compliant with splint utilization and did not regularly attend therapy. Despite her lack of compliance, she regained near full ROM within two months and full function within three months of her revision procedure.

## Discussion

Despite being rarely reported in the literature, we identified extensor tendon rupture as a complication in three out of 139 patients (2.2%) undergoing DRUJ procedures at our institution from 2000 to 2019. In the 1980s, three isolated case reports and two cases reported in a 29-patient series on the treatment of distal radioulnar disorders separately reported extensor tendon ruptures following complete distal ulna excision due to the sharp edge of the distal ulna combined with distal ulna instability [13-16]. Nawijin et al. report one post-operative extensor tendon rupture in a series of 66 hemi-resection interpositional technique arthroplasties of the DRUJ while Wada et al. identified one extensor tendon rupture due to entrapment of the tendon between the repaired extensor retinaculum and distal ulna stump after a Sauvé-Kapandji procedure [6,17]. While causation is difficult to establish, the intra-operative findings in our series suggested that certain technical details during the initial procedure may have contributed to tendon rupture. For two patients, a sharp edge was noted on the distal aspect of the resected ulna, and for the third patient, there was capsular insufficiency. With the careful surgical technique, these causes could be minimized.

Aside from surgical technique, there may be other contributing factors. First, all patients who experienced this complication were, at minimum, in their seventh decade of life. While this would suggest that extensor tendon rupture following DRUJ procedures is more likely to occur in elderly patients, it is more likely secondary to the characteristics of the patient population undergoing DRUJ procedures at our institution. Second, all three patients who experienced extensor tendon rupture had undergone a Darrach procedure. It is possible that there are aspects of this procedure that make patients more susceptible to this complication. In contrast to a Sauvé-Kapandji, the distal ulna resection for a Darrach procedure is more distal, which potentially puts the extensor tendons at greater risk. Moreover, during Bowers procedures, Sauvé-Kapandji, and DRUJ arthroplasty, there is greater soft-tissue preservation, which may afford some increased distal ulna stability. However, this complication has been reported to occur in all of these procedures [6,17,18].

The present case series highlights a few important points of consideration. Following intra-operative completion of the distal ulna resection, the surgeon should carefully inspect for any prominences that may cause irritation to extensor tendons and ultimately attritional rupture. Furthermore, attention should be directed to adequate capsular repair and/or reconstruction using local tissues. Finally, a careful examination of tendon motion should be performed by passively manipulating the hand and wrist to see how these tendons move under direct observation. Doing so may help identify any sources of irritation that are not readily apparent on initial examination.

Overall, as evident by our three occurrences over a 19-year period, extensor tendon rupture following a DRUJ procedure is a rare complication [13,14,17]. Surgeons may be able to minimize the risk of this complication intra-operatively by ensuring that the resected distal ulnar stump is smooth, that the DRUJ is free of bony prominences, that any capsular deficiencies are reconstructed, that the distal ulna and DRUJ are stabilized intraoperatively, and that extensor tendons are able to move freely. Nonetheless, even with attention to these details, this complication may still arise. Therefore, it is important for physicians to inform patients of this potential complication in order to better guide their decision-making.

## Conclusions

DRUJ arthritis can be a source of substantial pain and functional impairment that may be surgically managed with distal ulna resection in combination with tendon reconstruction. Extensor tendon rupture as a complication of distal ulnar resection rather than DRUJ arthritis is rare, with three isolated case reports in the past 30 years. We identified three patients that developed attritional ruptures after distal ulna excision due to sharp prominent bone edges or dorsal capsule disruption. Surgeons may be able to minimize the risk of this complication intra-operatively by ensuring the resected distal ulnar stump is smooth, free of bony prominences, any capsular deficiencies are reconstructed, and that extensor tendons are able to glide freely.
